# Identification and Speed Estimation of a Moving Object in an Indoor Application Based on Visible Light Sensing of Retroreflective Foils

**DOI:** 10.3390/mi12040439

**Published:** 2021-04-15

**Authors:** Andreas Peter Weiss, Franz Peter Wenzl

**Affiliations:** Institute for Surface Technologies and Photonics–Smart Connected Lighting, Joanneum Research Forschungsges mbH, Industriestrasse 6, A-7423 Pinkafeld, Austria; franz-peter.wenzl@joanneum.at

**Keywords:** visible light sensing, retroreflective foils, photonic sensors

## Abstract

Identification and sensing are two of the main tasks a wireless sensor node has to perform in an Internet of Things (IoT) environment. Placing active powered nodes on objects is the most usual approach for the fulfillment of these functions. With the expected massive increase of connected things, there are several issues on the horizon that hamper the further deployment of this approach in an energy efficient, sustainable way, like the usage of environmentally hazardous batteries or accumulators, as well as the required electrical energy for their operation. In this work, we propose a novel approach for performing the tasks of identification and sensing, applying visible light sensing (VLS) based on light emitting diode (LED) illumination and utilizing retroreflective foils mounted on a moving object. This low cost hardware is combined with a self-developed, low complex software algorithm with minimal training effort. Our results show that successful identification and sensing of the speed of a moving object can be achieved with a correct estimation rate of 99.92%. The used foils are commercially available and pose no threat to the environment and there is no need for active sensors on the moving object and no requirement of wireless radio frequency communication. All of this is achievable whilst undisturbed illumination is still provided.

## 1. Introduction

The Internet of Things (IoT) has become one of the main buzzwords in recent years, which describes the interconnection of objects—both real physical objects as well as virtual objects—by the means of various communication technologies in order to form a widely distributed network, which shares information to perform an ever increasing number of tasks and applications. The origin of the term, as well as the technological foundations, emerged in the late 1990s. Several researchers and scientists outlined the vision of a massive interconnection of objects via the Internet in this decade. The specific term, IoT, anyhow, is nowadays attributed to Kevin Ashton [1]. In the early years, this term strongly referred to the usage of Radio Frequency Identification (RFID) to perform tasks like identification and tracking [1], but already in these early years, the concepts were designed to involve various technologies and communications concepts. In [2] the elements of an IoT are described as identification, sensing, communication, computation, services and semantics. Whilst services and semantics refer to tasks that usually reside in higher layers of such an IoT framework, for example, in a Cloud computing environment, identification, sensing and communication can be seen as the lower levels of such a network [3]. Identification is the task of uniquely determining various objects that are aimed to be recognized in an IoT environment. The task of sensing describes the acquisition of data about the object, which, in combination with the unique identifier, are finally communicated wirelessly. Many technologies have evolved over time to be used in the context of IoT to perform the task of communication, such as Wi-Fi, Bluetooth, RFID and many more [4]. Processing the acquired readings in order to extract higher-level information or to create the context of the object is summarized in the task of computation. In most cases, in order to perform the described tasks of identification, sensing and communication, active devices—often referred to as Wireless Sensor Nodes (WSN) [5]—are placed on the object. The architecture and components used to form these nodes are strongly dependent on the application and services to be performed and consequently have reached a vast range of possible configurations and capabilities, ranging from small and simple devices, for example an RFID tag [6], to complex wearable devices such as smartwatches and so forth [7].

The increase in connected IoT devices worldwide is ongoing and is building momentum. Statista [8] forecasts over 30.9 billion connected devices by the year 2025. With this massive increase on the horizon, there are many challenges emerging, ranging from energy consumption and electronic waste to bandwidth limitations [9,10]. The International Energy Agency estimates the power used by IoT devices by 2040 to be more than 2000 TWh [11]. To reduce this energy consumption many solutions have been proposed, which range from utilizing extended standby modes in the WSN to energy saving routing schemes [12]. Active components on the WSN require some kinds of batteries or accumulators. In order to provide the longest possible runtime of the node, energy saving schemes as well as energy harvesting technologies have evolved [13]. Even with extended runtime, the problem of electronic waste concerning IoT devices is still unresolved [9]. The accumulators and batteries, which are often not easily detachable from the device, especially lead to the problem of an increasing number of devices that require special treatment in the recycling or disposal processes. As stated in [10], even in case that changing the batteries is technically possible, the large number of nodes makes this process unfeasible and results in a throwaway mechanism at the end of the node’s lifetime. With the widespread use of actively communicating devices, even in cases where the communication demand is kept minimal, the bandwidth of the used communication technologies is anticipated to become a bottleneck [10]. Furthermore, in cases where the number of devices increases, as all forecasts suggest, the interference between wireless Radio Frequency (RF) based communication technologies is anticipated to become an issue. To enable the further widespread of the IoT and in order to provide solutions for the future massive increase in connected devices, applications and services, innovative solution approaches are indispensable.

The advancements in the area of LEDs, photosensitive devices and their corresponding electronic components and circuits have led to several technologies based on the utilization of visible light. One of the most prominent one is the wireless transfer of data in the visible light spectrum by modulating the intensity of a light source and consequently demodulation at a photosensitive receiver to recover the transmitted data. This application is referred to as Visible Light Communication (VLC) [14]. Another application, realized on the base of light emitted from a luminaire, is called Visible Light Positioning (VLP). With the help of VLP, a human or an object equipped with a VLP receiver unit, usually consisting of a photosensitive device (e.g., photodiode (PD)), associated electronic circuits (e.g., Transimpedance Amplifiers (TIAs)) and a processing unit, can be localized by measuring and analyzing the impinging light on the receiver unit [15]. The technology of VLS is more or less a general term describing various tasks on the basis of extracting information from the received light intensities and in some cases from the spectral composition of the light at a photosensitive device. The applications range from occupancy or presence detection [16,17], identification [18], pose detection [19] to gesture recognition [20]. Similar to this broad range of applications, the applied devices, the orientations of the light sources and the receivers in relation to each other and the used algorithms also vary largely. In [21] the scenarios of VLS are divided into four distinct categories. The main distinction is done by separating Line of Sight (LoS) and Non Line of Sight (NLoS) scenarios and also separating scenarios in which some form of modulation of the light is performed or not. In this work, we will be dealing with a scenario in which an object reflects the light emitted from the light source towards a photosensitive receiver. For this study we assume that the light source itself is not modulated, but the object reflecting the light towards the receiver carries retroreflective foils, which modulate the intensity and the spectrum of the reflected light according to the colors and the size configurations of the attached foils.

In [22], it is shown in an outdoor scenario that, by placing a pattern of black and white foils on one side of a car, it is possible to decode this pattern successfully over a distance of 2.5 m to 4 m with only natural sunlight as the light source. The car was passing the receiver unit with speeds of up to 53 km/h. The results show that the concept of VLS can be applied successfully for moving objects. The key differences to the approach we are discussing in the following are that in an outdoor scenario the size of the reflective area is not constrained as strongly as in our indoor application scenario. Second, the reported light intensities in the outdoor scenario—~3 kLux (cloudy day) and ~10 kLux (sunny day)—are also far beyond the applicable light levels for an indoor scenario. Third, the work reported in [22] was limited to “tags” consisting of black and white areas, whilst leaving out the possibility of also incorporating the spectral composition of the reflected light into the algorithm in order to generate a higher degree of freedom of possibilities for the variation (coding) of the reflective “tags”.

Indoor positioning and the identification of objects equipped with reflective tags is discussed, for example, in [23]. In this work, a set-up consisting of multiple luminaires placed on the ceiling of a room, which act as the light sources, a complex receiver system placed besides them and reflective tags on objects, is discussed in a theoretical/simulation based approach. The reflective tags consist of multiple stripes of a reflective material that reflects the light in a very narrow bandwidth. In parallel to the tag design, the receiver system is also designed to incorporate photodiodes that have a very narrow sensitivity range (~2 nm). In the simulation of the hardware design, the receiving photodiodes were tuned with the help of a laser diode to this narrow sensitivity range. Once the photodiode was tuned, it was sensitive to only the reflection of one of the stripes of the tags and recorded this peak value. Then the photodiode was tuned to the next sensitivity range and the peak value of the next stripe was recorded, and so forth. On the basis of a continuous tuning, it was calculated that 2^150^ − 1 different tags could be identified. With different simulated grid sizes, the achieved positioning error was reported to be in the range between 2 cm and 35 cm. This work demonstrates how highly accurate positioning and identification tasks can be performed by utilizing the spectral variation of the reflected light, however, to do so, very narrowband reflective tags and highly tuned photodiodes are necessary. This causes a system of high complexity, which will require customized components such as the reflective tag stripes. This is in strong contrast to our work, where we use off-the-shelf components and a low complex hardware design in our experimental setup. Additionally, the object was simulated as stationary during the acquisition period and not as moving. As we will show in the following, the movement of the object and consequently its position in relation to the sender–receiver system will strongly influence the intensity and composition of the reflected light. This behavior will be one of the main characteristics for the determination of the speed of the object itself. 

Another interesting work in the field of VLS is given in [24], where by placing reflective materials (mirrors or aluminum) on a toy car, the authors show that the localization of this toy car can be achieved by exploiting the reflected light in a scenario with multiple luminaires illuminating the scenario. Each of the light sources in this work modulated the respectively emitted light to forward its unique ID. Based on the received reflected light on multiple receivers, a determination of the position could be achieved. Furthermore, the authors expanded the application scenario towards the identification of the single toy car by placing a pattern, comprising of strongly reflective areas and strongly absorbing (black) areas, on the car. With the main tasks of determining the position of the moving object and its identification, this work shows how the technologies of VLC, VLP and VLS can be realized in parallel without impeding each other. In contrast to our approach, the speed of the object had to be calculated from the estimated positions and was not directly inferred as in our approach. Also, the applicable number of different IDs is limited for such a configuration, because of the size requirements for the strongly reflective and the strongly absorbing (black) areas.

In our previous work [18], we showed how, by placing different reflective foils on the six sides of a small static cube, the different foils could be determined successfully by utilizing a single RGB sensitive photodiode and a single LED light source. This previous achievement of determining different reflective foils in a static setting is the starting point for the expansion of the application scenario to a moving target in this study. The expansion of the range of the applied foils and furthermore to take different size configurations of the reflective areas into consideration are done to provide an extensive platform for the coding of moving objects in order to identify them and to determine their speeds. 

In particular, we will show how, by utilizing this platform for VLS, one can perform the described tasks of identification, sensing and (backscattered) communication, required in the lower levels of an IoT environment, without the need for any active components, which again would require a battery or an accumulator placed on the object. This can relieve some of the challenges anticipated for IoT. Furthermore, by utilizing the visible light spectrum, the described tasks can be performed solely on the basis of the already existing lighting infrastructure; therefore, no additional communication components—for example, Wi-Fi Access Points—have to be placed in the infrastructure. In contrast to batteries and accumulators, the used reflective foils do not contain any environmentally hazardous materials and therefore can be easily disposed when necessary. By utilizing the visible light spectrum, existing RF based communication systems can also be relieved from the communication effort these tasks would cause, if they would be carried out in the RF spectrum. 

This article is divided into the following sections. In Section 2, Materials and Methods, the used materials and the experimental setup as well as the implemented algorithm are described. In Section 3, the results of the experiments, both for the task of identification as well as for the combination of the tasks of identification and sensing the speed, are presented. Section 4 describes possible simplifications that could be undertaken in order to reduce the demands for a future microcontroller unit (MCU) or field programmable gate array (FPGA) based implementation. Section 5 finally summarizes and discusses the achieved results and outlines future research challenges and directions.

## 2. Materials and Methods

This section is subdivided into 4 subsections, first the used electronic hardware (the VLS unit) is described. Second, the utilized retroreflective foils of the vendors 3M and Orafol are specified. In the third subsection the experimental setup is given. Finally, the implemented algorithm as well as the conducted experiments are outlined.

### 2.1. VLS Unit 

**Figure 1:**
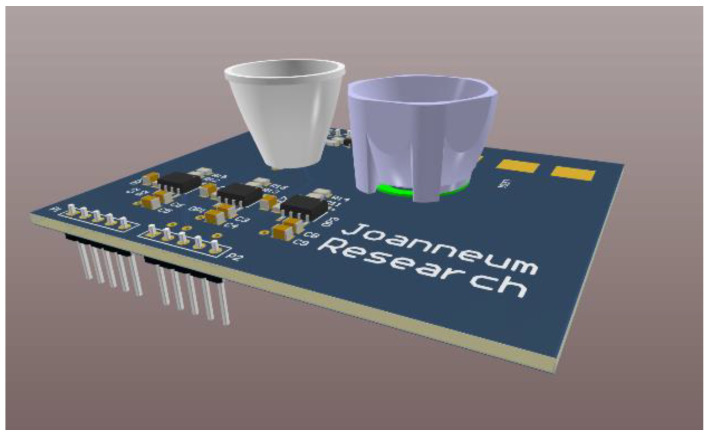
3D model of the VLS unit.

The main block of our experimental setup is our self-developed VLS unit with its CREE MC-E LED [25] as the light source and the RGB sensitive photodiode Kingbright KPS-5130PD7C [26] as the sensing device. The RGB sensitive photodiode has one common cathode and three anodes corresponding to the channels (R, G, B). The three channels of the photodiode are interfaced to three separate TIAs, transforming the currents induced by the impinging light at the corresponding channels into voltage signals. Such a voltage signal can then be easily sampled with an Analog-to-Digital converter (ADC), transforming it into the digital domain. Please note that the design of the TIA was done in a way that a lower voltage signal at the TIA output corresponds to a higher amount of impinging light. Therefore, a value of zero at the TIA output corresponds to a saturation of the photodiode. The used Cree LED consists of four separate white dies, which are all connected to the same input pin, providing the electric supply. Although the VLS unit also incorporates a Field Effect Transistor (FET) driver to allow for a modulation of the LED, in this work we did not modulate the LED. Furthermore, to achieve better directionality of the emitted light of the LED, as well as of the impinging light on the photodiode, we placed off-the-shelf reflectors over the LED as well as over the photodiode. The reflector placed over the LED is a CA10928_BOOM of the vendor Ledil that offers an optical efficiency of 90% with 36 degrees full width at half maximum (FWHM). The reflector over the photodiode is a Ledil C11347_REGINA with a ~9° spot beam. Further details can be found in [18,19]. Figure 1 shows a 3D model of the VLS unit, showing the LED with the reflector (right) and the reflector placed over the photodiode (left). The supply voltage of the TIA circuitry is 5 V, with a current of ~11.39 mA.

### 2.2. Retroreflective Foils

A material is considered retroreflective, when it is reflecting radiation—in our case the light—back to its source with minimal scattering. The application range of such materials is broad, but probably the best-known application example in the visible light spectrum are traffic signs. By placing retroreflective foils on the traffic signs, the incoming light from the car is mostly reflected back towards the car and therefore can be seen by the driver. In the following, we utilize the retroreflective characteristics of different colored foils. As shown in Figure 1, the light source and the receiving RGB photodiode are placed closely together on the VLS unit so that, by exploiting the attribute of retroreflection, one can assume that the light emitted from the LED is mostly reflected backwards to the receiving element, the RGB photodiode. Retroreflective foils were also chosen since they are available in a broad range of colors as well as in different setups, like the ones from the vendors 3M and Orafol. These foils are usually available as sheets of variable lengths and have an adhesive backside, so that they can be placed very easily on objects. Overall, the utilized foils are very easy to handle since they can be easily cut to the desired sizes and shapes. This is one of the main advantages of such foils compared to other materials such as (colored) mirrors. Five of the differently colored foils that were used for the study were purchased from the vendor 3M (production family 4000 [27]). These colors and their respective production codes are given in Table 1.

From the vendor Orafol three different colors of the VC170 family [28], given in Table 2, were used. 

**Figure 2:**
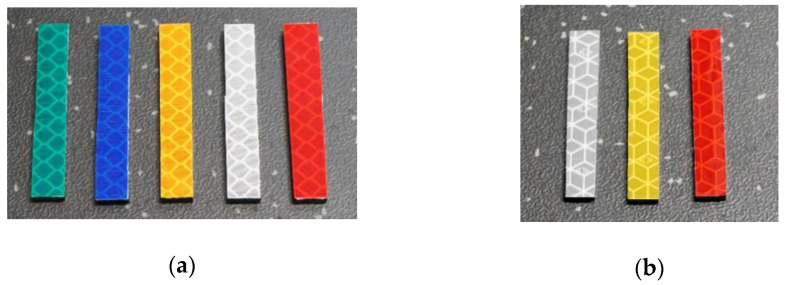
The used retroreflective foils in a size configuration of 0.7 cm × 4.7 cm, (**a**) 3M retroreflective foils (**b**) Orafol retroreflective foils.

The internal buildup of the foils from both vendors is based on light-guiding microstructures to achieve the retroreflective characteristics. Figure 2 shows the used foils, with Figure 2a showing the five 3M foils, whilst Figure 2b shows the three Orafol foils.

Within the experimental setup, described in the following subsection, these foils were placed on a moving object in different size configurations. 

### 2.3. Experimental Setup

The moving object in the experimental setup is based on a LEGO train (60197 LEGO City), which was adapted according to the requirements. Whilst leaving only the control block, the motor and the wheels with its connected platform in its original state, the power supply was changed to a rechargeable accumulator with a DC/DC converter to guarantee a more stable supply, compared to the original battery based supply, and much longer runtimes of the train. The body of the train is made up of black components to form a cuboid, which is 22.3 cm in length, 4.7 cm in width and 8 cm in height. On top of the cuboid, the retroreflective foils were placed in the center of the platform. In order to allow for an easy installment and removal of the foils, the foils with dimensions of 0.7 cm × 4.7 cm were placed with the adhesive side on black LEGO bricks. The upper side of these utilized LEGO bricks is plain, whilst the lower side has the known LEGO connection. The different size configurations of the reflective areas were built-up by placing up to four of these LEGO bricks beside each other. In our experiments we varied the sizes of the reflective areas from the smallest dimension of 0.7 cm × 4.7 cm (1 brick) to the largest dimension of 2.8 cm × 4.7 cm (4 bricks), in increments of 0.7 cm. In the following, we will use the names of Area 1–0.7 cm × 4.7 cm, Area 2–1.4 cm × 4.7 cm, Area 3–2.1 cm × 4.7 cm and Area 4–2.8 cm × 4.7 cm for these different dimensions.

**Figure 3:**
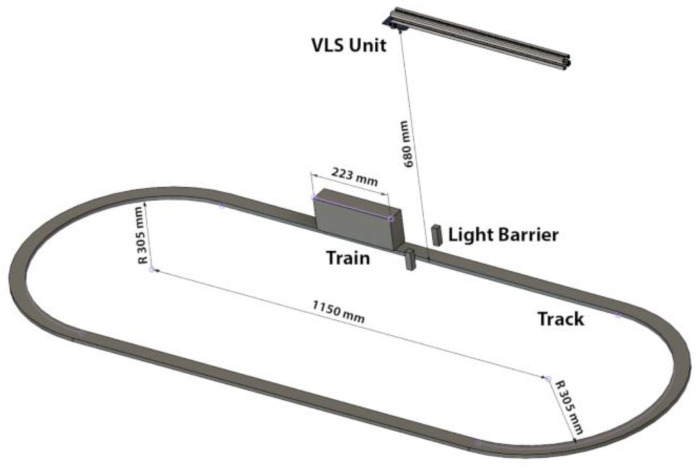
Sketch of the experimental setup, showing the track and the placement of the VLS unit.

The greyish colored plastic rails, on which the train is moving, were set up to form a track that resembles the number zero. The straight parts of this track are 115 cm in length. The VLS unit was placed on a metallic bar facing downwards towards the rails over one of the straight parts of the tracks. By this, the LED and PD are placed over the center width of the rails with a distance of 68 cm between the VLS unit and the rails. Additionally, a simple self-developed infrared light barrier, consisting of a single infrared LED and an infrared sensitive photodiode, were placed alongside the rails under the center of the LED light spot. This light barrier was used as a triggering mechanism for the data acquisition. A detailed sketch of the experimental setup is shown in Figure 3.

The speed and the direction of the train were controlled via a wireless Bluetooth based controller unit. The direction of the train was kept constant throughout the experiments. The speed could be controlled in distinct steps. The chosen speed steps in the experiments are further on referred to as Speed 1, Speed 2, Speed 3 and Speed 4. The corresponding average velocities of the train are given in Table 3. 

Applying faster speeds was not possible due to limitations by the utilized train platform itself. But it can be outlined that the applied speeds are in good accordance with the reported speeds of robotic platforms such as automated guided vehicles (AGV) used in factory settings, for example, a max speed of 1 m/s for the Movexx AGV-Basic [29], autonomous mobile robots in healthcare, for example, TUG T2 with a max, speed of 0.76 m/s [30] or social humanoid robots such as pepper with a reported maximum speed of ~0.55 m/s [31]. This good analogy between the applied speed settings with the reported speeds of robotic platforms shows that these speed settings are of relevance for a future use of the approach to identify and estimate the speed of such robotic platforms.

**Figure 4:**
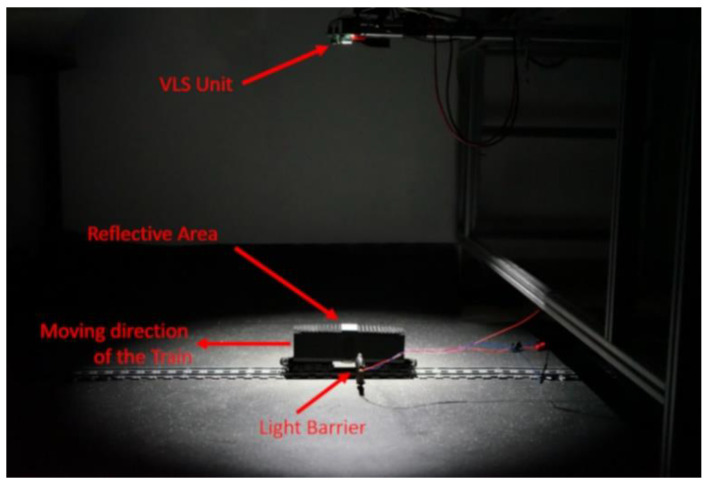
Experimental setup.

In the experimental setup the LED, the TIA circuitry and the light barrier were supplied via an external power supply. The LED was supplied with 3 V and 300 mA, resulting in ~690 Lux at the surface of the reflective area. The light barrier was operated with 3 V and 100 mA. Each of the three outputs of the TIA circuitry were connected to a channel of a Keysight DSOS404A Digital Storage Oscilloscope. The sample rate of the Oscilloscope was set to 5 MS/s. Figure 4 shows the experimental setup in our laboratory with the LED switched on and the labeling of the components, such as the train, the VLS unit and so forth. 

### 2.4. Algorithm and Conducted Experiments

In [18], we showed the successful classification of differently colored reflective foils placed on different sides of a small static cube with the VLS unit. In that work, we stored the different characteristic reflections of the separate foils to build a training set. In the testing phase, the actual impinging light on the photodiode and consequently the characteristic spectral composition was compared to the stored training set in order to determine the foil facing upwards, towards the VLS unit. In the scope of this work, this approach cannot be utilized in a straightforward manner since it is obvious that the received reflections will not be stable due to the movement of the object. In contrast to this previous work, the sizes of the reflective areas are also varied in the present study and a larger number of different foils is used. In order to handle these issues, we developed a new solution approach that is based on a time dependent training as well as assessment of the intensity and the spectral composition of the received light at the RGB photodiode.

In general, the designed algorithm consists of two phases—the offline (training) phase and the online phase. During the offline phase, the algorithm acquires the characteristic reflected values of the used foil in the respective size configuration and stores the output of the three channels of the photodiode circuitry over time. The acquisition starts when the train triggers the light barrier alongside the tracks and is fixed to a constant time period in which the ADC is acquiring the output values of the photodiode circuitry. In combination with the applied sample rate of the ADC; this results in a fixed number of samples that are acquired after the triggering event. After repeating the training runs for one known scenario (foil, size of reflective area and speed of the train) several times, a reference curve for this scenario is built-up. The generation of the reference curve of a scenario is described in the following paragraph. 

After finishing the selected number of runs for the scenario the reference curve should be built for, the output of the three channels of the photodiode circuitry over time is available for every of these runs. Each run has the same number of acquired samples from the ADC for each channel of the photodiode, starting with the index 1 (first acquired ADC sample after the light barrier has been triggered) up to the constant maximum number of samples. The constant maximum number of acquired samples results from the chosen time period after the triggering event, in which the ADC should acquire the output values, and of course the sample rate of the ADC. In the next step, the acquired values of the different runs are summed up, depending on their index. This means the sample with index 1 of run 1 is summed up with the sample with index 1 from run 2, the sample with the index 1 from run 3 and so forth. The sample with index 2 of run 1 is summed up with the sample with index 2 of run 2, and so forth. After the summation, the resulting dataset has the same number of values as a single run, with the difference that the values are now the sum values over all runs. In the final step of the reference curve built-up, every value of the dataset is divided by the number of runs. This results in a dataset that is consequently named a reference curve and which comprises of averaged values. 

To describe a scenario, we introduce a naming scheme, which will be used further on. The naming scheme always has the vendor of the respective foil at the beginning, 3M or Orafol. For the Orafol foils furthermore the abbreviation “O” is used instead of the full vendor name. After the vendor name, the color of the respective foil is given (e.g., red, blue, etc.). This is followed by the used size configuration, Area 1, Area 2, Area 3 or Area 4. At the end of the respective names, the speed of the scenario is given (see Table 3). So, for example if a scenario is named “3M blue Area 2 Speed 1”, this means that a blue 3M foil in the size configuration of 1.4 cm × 4.7 cm has been placed on the train and that the train was moving at a speed of ~0.68 m/s. The training is repeated for all selected scenarios in order to form a set of reference curves. In the online phase, in the first step, an unclassified dataset is compared to the different reference curves of each channel by computing the Euclidian distance of each sample point to the stored reference sample points. An unclassified dataset in this regard is a run of a scenario, which should be classified. For each available reference curve, the Euclidian distances between the sample points are computed. In the second step of the online phase, that reference curve, which yields the minimal sum value for the corresponding color channel, is reported as the classification result of this color. Consequently, in the third step of the online phase, a simple majority vote among the three color channels based classification results yields the final classification. If no majority can be achieved, the result is reported as undecided and consequently counted as a wrong classification. So, the output of the online phase will either be a classification result (the estimated scenario) or the report that the scenario cannot be estimated due to an undecided vote. The whole algorithm was implemented in GNU/Octave and was run on a conventional office laptop. In our current algorithm implementation, the data acquisition is started when the triggering event is initiated (train passes the light barrier). From the triggering event on, 2.5 million samples of all the three color channels were stored in a binary file format to form the respective dataset. The storing of the dataset to a file was done by a function of the Keysight oscilloscope, where automated trigger actions can be defined as soon as a triggering event has occurred. These 2.5 million samples correspond to 500 ms, which is a sufficient time frame for the train to move through the LED spot beam and the detection area of the photodiode for the chosen speed settings. The binary file format was chosen for an easy import in the GNU/Octave program.

The experiments in this work were conducted in one of our laboratory rooms, with the shades closed to completely block ambient sunlight. Furthermore, during the experiments we turned off the ambient light in this room, leaving only the LED of the VLS unit as the single light source. 

For the generation of the results presented in this work, we used the following workflow. The first step is the manual placement of the chosen foil with the selected size configuration at the specified location in the middle of the train platform. The second step, after placing the train on the rails, is to start the movement of the train with the chosen speed via the wireless control unit. When the train crosses the light barrier, the first dataset is stored, when the train crosses the light barrier again, the second dataset is stored and so on. Once a sufficient number of datasets for this configuration of the foil and the selected speed has been recorded, the workflow is repeated beginning again with the first step of placing the next chosen foil with its selected size configuration on the train, until all of the potential scenarios are recorded. In our experimental setup, we conducted 20 runs for each scenario. The thereby created files are numbered with indexes ranging from 1 to 20. These files are transferred to the laptop for the execution of the algorithm. For the algorithm execution and assessment of the performance, the available 20 datasets for each scenario are split into 10 datasets that generate the reference curves and into 10 datasets that are used as the online phase of the algorithm as the unclassified datasets. Since the correct scenario is known for every dataset used in the online phase, it can be easily assessed whether the output of the online phase is correct or not. All datasets with an even number as an index are used to generate the reference curves, whilst all files with an odd number as an index are used as online datasets. 

**Figure 5:**
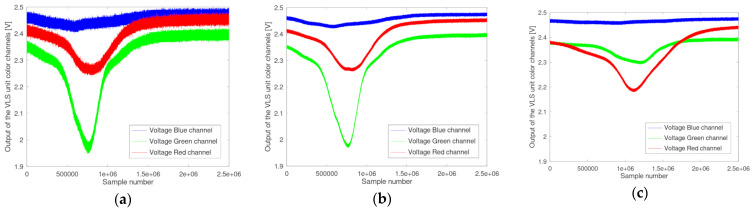
(**a**) Single run of scenario “O yellow Area 4 Speed 4” (**b**) computed reference curve of scenario “O yellow Area 4 Speed 4” after 10 runs (**c**) computed reference curve (10 runs) of scenario “3M red Area 4 Speed1”.

Examples of such generated data are shown in Figure 5, where on the left side (a) the data for one single run of the scenario “O yellow Area 4 Speed 4” are shown. In the middle (b) the computed reference curve for this scenario after 10 runs is given. On the left side (c) the computed reference curve of a different foil at a different speed level, namely the scenario “3M red Area 4 Speed 1”, is shown. The x-axis shows the sample number, whilst the y-axis shows the measured output voltage of the corresponding color channel.

**Figure 6:**
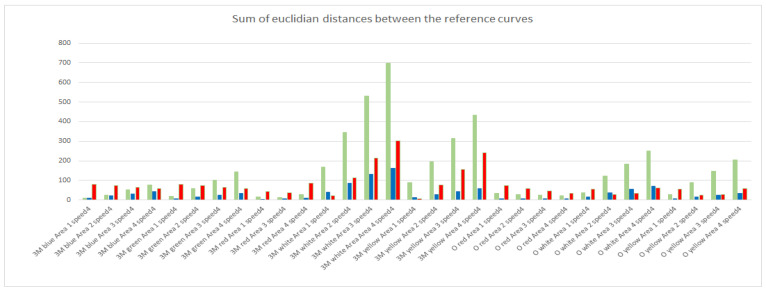
Sum of Euclidian distances between the scenario “3M red Area 2 speed 4” and all the other scenarios for the same speed level.

From the curves shown in Figure 5a–c, several conclusions can be drawn. By comparing Figure 5a to Figure 5b, it becomes obvious that by accumulating and building the average over 10 runs, the noise level in the generated reference curve can be reduced. The comparison of Figure 5b to Figure 5c demonstrates how the speed of the moving object is affecting the reference curve. Whilst in Figure 5b, the reflective area is leading to a distinct change in the spectral composition of the impinging light after ~500,000 samples (~100 ms) after the triggering event (Sample 1), at a lower speed as shown in Figure 5c, this change can be observed at ~800,000 samples (~160 ms). Analyzing the reference curves of the scenarios can furthermore give a detailed insight into the differentiation between the scenarios. This can be achieved in a similar way as to that described for the online phase of the algorithm, by computing the sum values of the Euclidian distances. For every sample in the reference curve, the Euclidian distance to the corresponding sample of a different reference curve can be computed. By applying this to all samples between the two reference curves and building the sum, a measure of how similar these two reference curves are is derived. In the following example, the reference curves of all possible scenarios at a distinct speed level were built. Since the different scenarios include all in all 8 different foils, each of them with 4 size configurations, this will lead to 32 different scenarios for this speed level. Therefore, by building the sum value of the Euclidian distances of one scenario against all the remaining scenarios, a vector of 1 × 31 is generated. Building this vector for every scenario results in a 31 × 31 matrix containing the sums of Euclidian distances between the scenarios. By choosing one particular scenario (row of the matrix), it can be investigated how well this scenario can be distinguished from the other scenarios (columns of the matrix). Figure 6 shows an example for the scenario “3M red Area 2 Speed 4” as a bar chart, where the y-axis is the absolute number of the sum of the Euclidian distances for the respective color channels (shown with their respective color), against the other scenarios with the same speed setting, given on the x-axis. 

**Figure 7:**
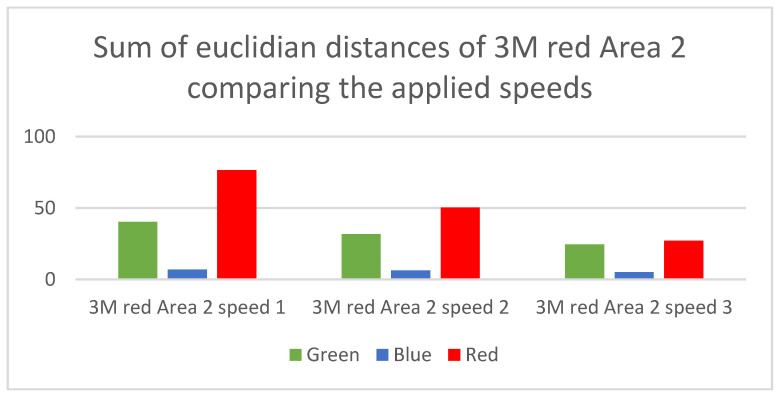
Sum of Euclidian distances between the scenario “3M red Area 2 speed 4” and the scenarios with the other speed levels for this foil and size configuration.

In Figure 6, it is clearly observable that the most distinguishable scenario is “3M white Area 4 Speed 4”. This is based on two facts. First, the white foil reflects the impinging light for the whole spectrum and second the reflective area has the double size, which leads to a higher intensity of the reflection. Another observation that can be made is that the spectral composition of the reflected light leads to a significant distinguishability between the colored foils. For example, the 3M blue foil differs the most from the 3M red foil in the signals received in the red channel. Additionally, scenarios that have very little sum values in one or two color channels can also be identified. These scenarios are more likely to cause misclassifications since the reference curves in the respective color channels are more similar. Another possibility is to generate the comparison regarding the different speed levels of the train. With this comparison a good insight can be generated about how distinguishable the speed levels are. Figure 7 shows the bar chart for the scenario “3M red Area 2 Speed 4” compared to the other three applied speed levels of this scenario, with the same representations as in Figure 6, where the y-axis gives the absolute number of the sum of the Euclidian distances for the respective color channels. 

Figure 7 shows the influence of the speed on the Euclidian distance. It is clearly observable that the larger the speed difference, the better is the distinguishability. This is a clear consequence of the time-dependent difference of the reference curves, as can be seen from comparing Figure 5b and Figure 5c.

Overall, this comparison, based on the reference curves, can be used in future work in more critical and challenging environmental settings, for example, lower illumination, to pinpoint scenarios that will still be distinguishable. 

## 3. Results

We started to generate the results that determine the amount of correct classifications of the scenarios by training and testing scenarios with the same speed. As described in the previous section, 20 runs per scenario for four speed levels of the train were performed. If one considers scenarios with the same speed, this leads to overall 32 reference curves and 320 test cases (10 per scenario). 

The results for Speed 1 show that all 320 test cases are determined correctly. Only in 3 runs of the scenario “O yellow Area 2 Speed 1” the decision was not reached by having three out of three but having two out of three. 

The results for Speed 2 still yield all determinations as correct. For Speed 3, the correct determination rate is also 100%. At the highest speed level, Speed 4, the results show that 4 determinations are incorrect. The incorrect results are two runs of the scenario “3M blue Area 3 Speed 4” and two runs of the scenario “3M red Area 2 Speed 4”. For all of these incorrect results, the classifications were marked as undecided. To recap, an undecided output means that no majority vote for the three color channels had been reached. With the overall 320 test cases, an incorrect classification of 4 test cases yields a correct estimation rate of 98.75 %. The reason why incorrect results only occur at the highest speed level is based on the much shorter period of time the reflective area is in the detection area of the VLS unit. As the train moves faster through the detection area, also the time period in which a meaningful reflection from the reflective area is received is much shorter, please compare Figure 5a to Figure 5c. Consequently, the amount of data points that allow for a distinction between the scenarios are less, since the last third of the samples of the reference curves are acquired when the train has already left the detection area. Nevertheless, these data points will play an important role in determining the speed of the train. Table 4 summarizes the results for the correct determinations of the scenarios for the different speed levels. 

Overall, these results show that our solution approach, utilizing retroreflective foils in different size configurations on a moving target, can perform the task of identification very reliably. Only at the highest speed setting, four incorrect classifications out of 320 occurred. Since these determinations are caused by an undecided vote of the three color channels and not a classification as a wrong scenario, we further improved the determination process for the case that an undecided vote occurs. 

### 3.1. Optimization of the Decision Process

For cases where the vote is undecided, a second level of decision-making was implemented in order to break the tie and to reach a decision. In this second level, the sum value of the Euclidian distances of the three estimates is the decisive factor. This means that, in a case where an undecided vote occurs, the color channel that yields the minimal value of the computed sum value to its chosen reference curves overrules the votes of the other color channels, and consequently this decision is then compared to the real scenario in order to determine a correct or incorrect classification. 

We applied this rule and reran the estimation at the highest speed level to determine the effect of this second level decision making. The results show that all four undecided results reach a correct estimation with this second level decision. The enhancement of the decision process improves the overall results to 100% correct identifications of all the scenarios at all speeds. 

### 3.2. Identification and Speed Determination

In the previous assessments, the main goal was to show the capabilities of the system for the task of identification. In this section, the task of performing the determination of the speed of the scenario under test is added. For this purpose, it is necessary to add all the available scenarios to the training dataset. This means that instead of having 32 reference curves the unknown scenario is compared to 128 reference curves (32 per speed). The unknown scenario is then compared to this reference curves by computing the sum of the Euclidian distances as described in the Algorithm description section, including the optimization of the decision process, as described in Section 3.1. We reused the same datasets as in the previous tests. 

Since 10 runs per scenario were used in the online phase, in total 1280 classifications were done during the online phase. A result is considered correct when the vendor of the foil, the color of the foil, the size configuration and the speed level as determined, all conform to the real scenario settings.

The results show that, except for one run of the scenario “O red Area 2 Speed 4,” all the classifications were correct. The scenario was misclassified as “O red Area 2 Speed 3”. A detailed investigation showed that, in this case, the foil and the size configuration were identified correctly, but the speed was misclassified. Since the speed of the train can be set to a certain level, but can vary within a specific margin, the misclassification happened because of such a variation, which led to the wrong determination. The fact that the speed of the moving object can also be determined solely by the received reflections of the used foils is achieved by the time depending comparison of the reference curve with the unknown scenario by the means of the sum of the Euclidian distances. As described earlier, the reference curves at higher speed levels tend to have no meaningful data after ~2/3 of the acquired 2.5 million samples, since the train has already left the main detection area. In comparison, at slower speeds in this time period still higher levels of reflected light from the foils are received. This difference causes that the sum of the Euclidian distances is high and therefore leads to the clear distinguishability of the scenarios. 

The achieved results prove that our solution approach provides highly accurate estimations and is capable of performing not only the identification task but also fulfills the sensory task of speed estimation of the moving object, without the need for any active component on that object. Table 5 summarizes the achieved results and the experimental setting. 

## 4. Implementation Considerations

The results show the general validity of our approach, but for an MCU or FPGA based implementation, the two following issues must be seen as critical:

1. Sample rate
2. Required memory size

In our experiments, we used a sample rate of 5 MS/s on the Keysight oscilloscope. Although the utilization of such high sample rates for an FPGA or MCU based implementation is possible, the design and proper operation requires a certain amount of expertise. From the thorough inspection of the attained data, we deduced that a reduction of the sample rate is possible without deteriorating the performance of the system. Utilizing a lower sample rate would furthermore follow our overall low complexity design approach. To show the effects of lowering the sample rate in direct comparison to the initially used 5 MS/s, we reused the same data as before, but resampled the signals, acquired by the oscilloscope and stored in the corresponding files, in the GNU/Octave software. 

In terms of required memory for the used implementation in Section 3, we acquired 2,500,000 samples per color channel. After building, the reference curves, these 2.5 million samples per channel must be stored in order to be available in the online phase of the algorithm. For the combined estimation of the foil and the size configuration alongside the estimation of the speed, we need to store 128 of these training datasets. Considering a 16-bit representation of the numbers, this results in a minimal memory requirement of the embedded processing unit of 640 MB. This memory must be accessible during the whole online phase of the algorithm continuously. Reducing the sample rate would also affect the required memory size directly since the amount of sample points of the reference curves will decrease.

**Figure 8:**
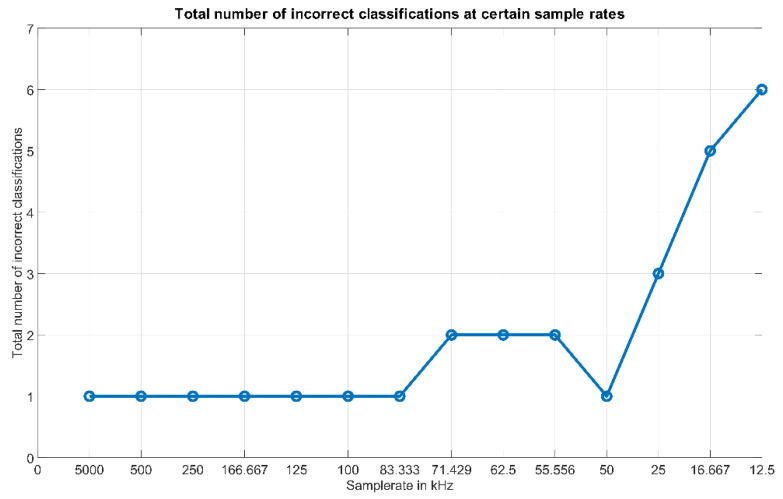
Total number of incorrect classifications at certain sample rates.

Figure 8 shows the total number of incorrect determinations (out of the 1280 test runs) in comparison to the applied sample rates, starting from the left side of the figure with the highest applied sample rate of 5 MS/s.

Figure 8 shows that, with a reduction of the sample rate down to ~83 kHz, the same good results with regard to the tasks of identification and speed estimation can still be achieved. Below this value, the results start to become volatile, which can be observed by the fact that the number of incorrect classifications increases to 2 at 71.429 kHz, but at 50 kHz this number drops again. The reason for this behavior is due to the fact that at these low sample frequencies only an insufficient number of data points is generated. At these low rates, the outcome of the reference curve generation and the online test is basically affected by chance, whether the acquired data points map the course of the curves detailed enough or not. If it is not detailed enough, this will deteriorate the distances between the scenarios and therefore also deteriorate the performance of the algorithm. As already mentioned, if at a certain sample rate (see 50 kHz) this detail is acquired by chance, the algorithm still can distinguish the scenarios. Since such a contingency must be avoided in order to guarantee a stable output, the sample rate with the chosen speed of the object should be kept at 100 kHz or higher. Further analysis of the results for the “volatile” sample rates shows that down to 25 kHz only the scenarios with the highest speed levels are affected, whilst the performance regarding scenarios at lower speed levels remains stable. The reason for this is obvious—at lower speeds the curves peak values span over larger time periods and therefore even at lower sample rates the characteristics of the curves are mapped out correctly. Below 25 kHz, also scenarios at lower speed levels are affected. This leads to the possibility that when the speed of the object is known to be low, the sample rate can also be decreased further.

Regarding the speed of the object, it was observed that at 100 kHz and above the highest speed of ~1.06 m/s has no negative effect on the results. It must be noted that a further increasing of the speed level of the object will cause more incorrect determinations due to the shorter time period the foils are reflecting light towards the photodiode. This negative effect will be even the larger the lower the applied sample rate is. Especially when the size of the reflective area is small, more incorrect results must be anticipated when the speed of the object is increased above the value of ~1.06 m/s applied in the experiments.

To relate these findings to the considerations regarding the necessary sample rate and the required memory size, one can draw the conclusion that by reducing the sample rate to 100 kHz, the design and operation of such ADC circuits is unpretentious and also the components in this range are cheaper. For the required memory size this means that, at 100 kHz, one reference curve consists of 50,000 samples. If we apply the same 16-bit representation, this would lead to an overall memory requirement of all the reference curves of only 12.8 MB instead of 640 MB. This expands the range of applicable processing units and also the components can be expected to be cheaper. 

## 5. Discussion

In this study, we investigated a system that can perform the tasks of identification and speed estimation of a moving object, which solely rely on the assessment of reflected light from different retroreflective foils placed on the moving object. We showed that, by employing an algorithm approach that is based on reference curves to describe a certain scenario (type of foil, size of reflective area and the speed of the object) and relate an unknown scenario to the established reference curves, not only the foil but also the size of the foil and the speed can be determined with highest accuracy. The mathematical method for establishing the mapping and consequently computing a robust decision parameter is based on the computation of the Euclidian distances between the reference curves and the unknown scenario. By applying this method, a time dependent measure of how well a reference curve and the unknown scenario match can be generated. Building the sum value of the Euclidian distances finally generates a single parameter for each color channel that yields the best match for the unknown scenario. 

The experimental results prove that this method results in a large number of correct estimations, with only 1 misclassified scenario out of 1280 tests. In the study, we utilized eight different foils in four different size configurations, which results in 32 distinguishable markers that can be placed on an object. The applied speeds of the moving train are in good agreement with the speeds of robotic platforms such as AGVs, and so forth. Therefore we would like to outline that our system can be of direct relevance in settings where a mobile robot needs to be identified, without the need for any active communicating components (e.g., Bluetooth or Wi-Fi), by simply placing the foils on the robot and performing the necessary training. Besides the identification and differentiation of different robots (e.g., one wears reflective foils of larger size and the other reflective foils of smaller size) and their speeds, it is also feasible that the moving direction (forward, backward) should be differentiable, simply by the sequence of differently colored foils (e.g., blue follows red for a forward movement while for a backward movement in this case red follows blue). One of the limitations of our approach is the way the object passes through the detection area, which in our setup is given by the rails the train moves on. For future work, it will be necessary also to investigate scenarios where the paths through the detection area can vary. Nevertheless, in current real world applications like autonomous mobile platforms in factory or warehouse settings, the number of paths a robot moves on is also limited, thus again showing similarities with our proposed setup.

In the present study, we focused on the task of identification and speed estimation of the object in the absence of ambient light. In order to outline how this limitation could be overcome, we would like to refer to [18,32]. In [18] we showed how, by a simple correction step, the influence of artificial ambient light (fluorescent tubes) can be compensated for by recording the changed ambient conditions for only one scenario and inferring the necessary compensation. This compensation can be applied to the trained scenarios, without the need for a complete retraining of the system. Another possibility to deal with ambient light is not to utilize the raw values from the photodiode for the corresponding channels, but to compute relative parameters describing the impinging light on the photodiode. In [32] we investigated the determination of the direction of a rotation performed by a robotic arm, based on visible light sensing of retroreflective foils placed on the robotic arm. In [32] we utilized the same foils as in this work, with the difference that not the raw values were utilized but rather relative values. The utilized relative parameters are the ratios of three channels against each other (Red/Green, Red/Blue and Green/Blue) and the difference between the channels (Red–Green, Red–Blue and Green–Blue). The experimental results showed a good resilience of these parameters against the influence of ambient light (switching on artificial lighting by fluorescent tubes in addition). Since in [32] the same foils were used, we can reason that this approach is also directly applicable to the application scenario discussed in this work.

One of the envisioned applications of our proposed system could be in a factory or warehouse with many autonomous mobile robots moving along certain tracks or paths. The number of moving objects that need to be identified along with their respective speeds will clearly be higher than the 32 different scenarios we showed in this work. In order to enable a higher number of distinguishable scenarios, three possibilities can be outlined. First, incorporating also foils with diffuse reflective behavior, since it can be anticipated that these foils will be clearly distinguishable from the retroreflective foils due to the changed intensity of the impinging light on the receiver, caused by the diffuse reflecting foils. Second, combining retroreflective foils of different colors, for example, 2.1 cm of red foil and 2.1 cm of blue foil. 

The third approach would be to increase the size of the area of one reflective foil beyond the maximum area of 2.8 cm × 4.7 cm used in this work. This is the most straightforward approach, but it must be noted that this solution can only be applied to a certain extent without further adaption of the system. In this work, we limited the timespan in which the object moves through the detection area and consequently in which the values are acquired by the photodiode. In a case where the size of the reflective area is largely increased, it is foreseeable that this timespan must be adjusted to generate a meaningful reference curve, since the algorithm relies on the fact that the reflections of the foil are also captured when the foil leaves the detection area. With the shown reduction of the requirement regarding the necessary sample rate, an extension of the timespan should not result in extensive additional computational resource demand, but still will increase the memory requirement. 

## 6. Conclusions

In our work, we showed that visible light sensing of retroreflective foils on a moving object can successfully achieve the tasks of identification and speed estimation of a moving object, without the need for placing any actively powered device or component on the object. The results show that, with our utilized low complex hardware and the implemented algorithm, stable and reliable results in real world experiments can be accomplished. The retroreflective foils are cheap, immediately available, easy to apply and do not include environmentally hazardous components or materials, as compared to batteries or accumulators used in actively powered WSN. Our presented solution approach is a good candidate to relieve the problems on the horizon, such as bandwidth saturation, e-waste or power consumption, regarding the expected massive increase in IoT devices and applications. Besides, all the required tasks can be taken over by the obligatory room lighting, which by exploiting LEDs for lighting, is a highly energy-efficient technology for itself [33]. 

By analyzing the requirements for an MCU or FPGA based implementation of our system, we were able to show further possibilities to reduce the demands regarding sample rate and memory size. Pursuing our investigations, in future works we will expand the scope of this work to enable the identification of more than the shown 32 scenarios (foil and size configuration), as well as the exploration of applying machine learning based approaches to support identification and speed recognition.

## Figures and Tables

**Table 1:** Colors of the retroreflective foils of the vendor 3M and the corresponding production codes.

Vendor	Color	Production Code
3M	Green	4097
3M	Blue	4095
3M	Yellow	4091
3M	White	4090
3M	Red	4092

**Table 2:** Retroreflective foils of the vendor Orafol with the corresponding colors and the production codes.

Vendor	Color	Production Code
Orafol	White	VC 170 #015
Orafol	Yellow	VC 170 #065
Orafol	Red	VC 170 #012

**Table 3:** Speed steps applied in the experiments and the corresponding average velocities of the train.

Speed Steps	Average Velocity
Speed 1	~0.68 m/s
Speed 2	~0.81 m/s
Speed 3	~0.94 m/s
Speed 4	~1.06 m/s

**Table 4:** Results of the scenario determination for the different speed levels.

Speed Level	Total Number of Test Runs	Absolute Number of Correct Determinations	Absolute Number of Incorrect Determinations
Speed 1	320	320	0
Speed 2	320	320	0
Speed 3	320	320	0
Speed 4	320	316	4

**Table 5:** Results of the combined execution of the identification and speed estimation task.

Total Number of Trained Scenarios	Total Number of Test Runs	Absolute Number of Correct Determinations	Absolute Number of Incorrect Determinations
128	1280	1279	1
